# Elementary math in elementary school: the effect of interference on learning the multiplication table

**DOI:** 10.1186/s41235-022-00451-0

**Published:** 2022-12-02

**Authors:** Dror Dotan, Sharon Zviran-Ginat

**Affiliations:** Mathematical Thinking Lab, School of Education and the Sagol School of Neuroscience, Tel Aviv University, 6997801 Tel Aviv, Israel

**Keywords:** Multiplication table, Proactive interference, Long-term memory, Math teaching methods

## Abstract

Memorizing the multiplication table is a major challenge for elementary school students: there are many facts to memorize, and they are often similar to each other, which creates interference in memory. Here, we examined whether learning would improve if the degree of interference is reduced, and which memory processes are responsible for this improvement. In a series of 16 short training sessions over 4 weeks, first-grade children learned 16 multiplication facts—4 facts per week. In 2 weeks the facts were dissimilar from each other (low interference), and in 2 control weeks the facts were similar (high interference). Learning in the low-similarity, low-interference weeks was better than in the high-similarity weeks. Critically, this similarity effect originated in the specific learning context, i.e., the grouping of facts to weeks, and could not be explained as an intrinsic advantage of certain facts over others. Moreover, the interference arose from the similarity between facts in a given week, not from the similarity to previously learned facts. Similarity affected long-term memory—its effect persisted 7 weeks after training has ended; and it operated on long-term memory directly, not via the mediation of working memory. Pedagogically, the effectiveness of the low-interference training method, which is dramatically different from currently used pedagogical methods, may pave the way to enhancing how we teach the multiplication table in school.

**Supplementary Information:**

The online version contains supplementary material available at 10.1186/s41235-022-00451-0.

## Introduction

Learning the basic arithmetic facts, in particular the multiplication table, is a key part of the elementary school mathematics curriculum. Mastering the multiplication table is important not only in itself but also for acquiring more advanced mathematical skills: even if not-memorized multiplication facts can be solved by various workarounds (strategies, external devices), automatic knowledge is still advantageous because it can free cognitive resources that can be used for other tasks (Bratina & Krudwig, 2003; Hasselbring, 1988). Sadly, learning the multiplication table is not only important, it is also difficult. Children typically learn by heart the single-digit multiplication facts from 3 × 3 to 9 × 9—a challenging quantity of 28 facts to remember. Other single-digit multiplications are not necessarily learned by heart, as they can be solved using rules, N × 0, N × 1, N × 10; using twin addition, N × 2; or using multi-stage procedures to solve multi-digit multiplication. Given this large memorization challenge, it is perhaps not surprising that many children have difficulties learning the multiplication table and show poor/abnormal performance patterns (Geary, 2004; Gross-Tsur et al., 1996; Noël & De Visscher, 2018; Räsänen & Ahonen, 1995).

Learning arithmetic facts, specifically the multiplication table, has at least two aspects. One aspect pertains to the mathematical meaning of arithmetic facts. The mathematical meaning determines the result of each given fact, and it has several consequences—for example, that addition facts are related to counting and to the idea of moving along a number line; that a multiplication fact is equivalent to a series of same-operand additions; and that for both additions and multiplications, larger operands are correlated with larger results. Learning such mathematical truths is critical to understanding the meaning of arithmetic and being able to use it properly. It can also help compute the result of arithmetic facts—e.g. if we need to solve a problem whose solution we did not learn yet or we forgot. Moreover, several of these truths are not just mathematical, they may also affect the cognitive processing of the arithmetic facts. For example, the magnitude of an arithmetic fact affects the difficulty of solving it (Groen & Parkman, 1972; Zbrodoff & Logan, 2005), and solving addition and subtraction facts is associated with the activation of number-line representations (McCrink et al., 2007; Pinheiro-Chagas et al., 2017).

The present study focuses on the second aspect of the knowledge of arithmetic facts, in particular multiplication facts—rote memory. This aspect is extremely important too: although arithmetic facts have mathematical meaning, and understanding this meaning is a critical stage of learning them, most educated adults eventually come to learn most single-digit arithmetic facts by heart, and they solve them by retrieving a memorized response, and not (at least not only) by applying mathematical rules (Campbell & Beech, 2014). More specifically, multiplication facts are stored in verbal memory (Dehaene, 1992; Dehaene & Cohen, 1995; Dehaene et al., 2003). In line with this idea, learning multiplication facts depends on language skills and memory (LeFevre et al., 2010; Xu et al., 2021; this is true also for arithmetic facts other than multiplication); and phonological skills predict arithmetic abilities (Jordan et al., 2010; Korpipää et al., 2020; Simmons & Singleton, 2008). Thus, while understanding the mathematical aspects of multiplication is necessary—it provides a way to solve multiplication exercises, and it underlies the knowledge of how to use multiplication for particular goals—rote memory helps become proficient in arithmetic. Indeed, in several countries, typical multiplication lessons include not only conceptual learning but also rote memorization of the multiplication table using various strategies, e.g., recitation or songs (Olfos & Isoda, 2021).

Because multiplication facts are stored as individual facts in memory, they are subject to the limits of human memory, in particular to the interference induced by the similarity between items: memorizing similar items is hard because they interfere with each other. This similarity effect is evident when learning arithmetic facts (Barrouillet et al., 1997; Campbell & Graham, 1985; De Visscher & Noël, 2013; Katzoff et al., 2020; Noël & De Visscher, 2018) as well as in other memory tasks (Baddeley, 1966a, 2003; Hall, 1971; Nelson et al., 1974; Oberauer & Kliegl, 2006; Oberauer & Lange, 2008; Stager & Werker, 1997; Vallar, 2006). Specifically for arithmetic facts, one explanation of the similarity effect is that the facts are represented in memory as a network of associations, in which each fact is associated not only with the correct solution but also with incorrect solutions (Campbell & Graham, 1985). The similarity between facts increases the likelihood of following these incorrect-solution associations and retrieving an incorrect answer. Some individuals have particularly high sensitivity to similarity-induced interference (“hyper-sensitivity to interference”), and consequently, they find it extremely hard to learn the multiplication table (De Visscher & Noël, 2013, 2014a; De Visscher et al., 2018; Dotan & Friedmann, 2019), i.e., they show symptoms of dyscalculia (American Psychiatric Association, 2013; World Health Organization, 1992).

As a means to overcome the difficulty caused by the similarity between facts, we propose a simple teaching method based on two foundations. The first foundation is *similarity versus dissimilarity*: similar multiplication facts are hard to memorize because they interfere with each other, but it may still be easy enough to memorize a set of dissimilar multiplication facts (Campbell, 1987; De Visscher & Noël, 2013, 2014a, 2014b; Girelli et al., 1996; Katzoff et al., 2020). Thus, learning the full multiplication table is hard, but it should be possible to learn a subset of multiplication facts as long as they are dissimilar from each other (e.g., 9 × 9 = 63 and 7 × 4 = 28). The second foundation is *temporal distance*: similar facts interfere with each other when they are presented simultaneously, or within a short time from each other, but interference should be lower when the facts are presented with a sufficient temporal delay between them (Campbell, 1987). Thus, we may be able to teach even similar facts (e.g., 8 × 8 = 64 and 8 × 6 = 48) if we present them with a sufficient temporal delay between each other. These two foundations lead to the following simple teaching method: each lesson includes only dissimilar facts, and different lessons that include similar facts are administered with a sufficient temporal delay between them.

We examined whether this low-similarity training method would improve the learning of multiplication facts by first-grade children who did not yet start learning the multiplication table. The specific experimental design was as follows: during four weeks of training, each child learned four multiplication facts per week (16 facts overall). In two weeks, the 4 facts were dissimilar from each other. As a control, in the two other weeks the facts were similar to each other. Thus, each child learned both similar and dissimilar facts. We predicted better learning in the weeks with dissimilar facts than in the control weeks. As we shall see, this was indeed the case.

In a previous single-case study (Dotan & Friedmann, 2019), this low-similarity training method was extremely successful for a woman with hypersensitivity to interference. A small effort of about 4 min per multiplication fact, distributed over 4 weeks, sufficed for her to learn 12 facts and to remember them two months later with 80% accuracy—much better than her performance in a high-similarity control condition. However, Dotan and Friedmann’s study had several limitations, which limit its ability to inform reliably about learning the multiplication table in common educational settings. First, the study examined only one participant, so its pedagogical conclusions are suggestive at best. Second, the participant was an adult woman, so the study does not inform directly about the memorization processes in children—the population that attends school and learns the multiplication table. Third, this woman had a very specific memory disorder (hypersensitivity to interference), so her performance pattern may be different from that of individuals without cognitive disorders, or individuals with other types of cognitive disorders. Last, although Dotan and Friedmann showed that low-similarity training can improve memorization, they did not examine in detail why this improvement occurred. For example, they did not identify the specific memory mechanism responsible for the improvement.

The present study aimed to overcome these limitations. We had three goals, which have an impact on pedagogy as well as on cognitive theory. First, we aimed to find a simple way to make it easier to learn the multiplication table for the most relevant population—typically developing children in early elementary school grades. Second, we aimed to show, for the first time, causal evidence for the effect of similarity between multiplication facts on their memorization, specifically in typically developing elementary school children. Third, we asked *why* similarity disrupts memorization; in particular, we aimed to identify the specific memory mechanism responsible for the similarity effect.

## Method

### Participants

The participants were native Hebrew speakers and were recruited via social networks. The inclusion criteria were that the child: (1) had no reported or suspected learning disorders, and (2) did not yet learn the multiplication table or the meaning of multiplication—neither before the study nor during the 12 weeks of the study.

35 children started the study. Additional file 1: Table S1 shows their details. Their ages were between 6;1 (6 years, 1 month) and 7;11 (mean = 7;1, *SD* = 0;5). They were in the first grade (27 children), 2nd grade (7 children), or the last year of kindergarten (1 child). Of these, 18 children were excluded. Three children were excluded immediately after the pre-experiment test because this test showed that they already knew some of the multiplication facts to be learned. Additional 15 children dropped along the way: 3 decided to quit; one was excluded for not following the study rules, which prohibited parental help; and 11 were excluded for being uncooperative or inattentive (for detailed exclusion reasons, and additional explanations, see Additional file 1: Table S2). Importantly, because we used a within-participant design, and each child performed both experimental conditions (low-similarity training and high-similarity training), the participant exclusions were not confounded with the experimental manipulation. Furthermore, the specific exclusion reasons were not related to the magnitude of a putative similarity effect: when we excluded a child based on objective measures, we never relied on a measure related to similarity, or on any performance measure reported in the results below; and when we excluded a child based on an experimenter’s impression of the child’s behavior in particular experiment sessions, the experimenters who made the decision were not told in advance which experimental condition was administered in those sessions.

These exclusions left 17 participants who completed the study and whose results are reported below (age range 6;1–7;11, mean = 7;0, SD = 0;5): two in second grade, 14 in first grade, and one in kindergarten senior year. None of them knew any of the 16 multiplication facts taught in the study (Additional file 1: Table S7a).

### Stimuli

Each child learned the same 16 multiplication facts, in which both operands were between 3 and 9. There were no ties (N × N). The smaller operand appeared first. The 16 facts were grouped into 4 sets with 4 facts in each: two sets with low similarity among the facts, and two sets with high similarity (i.e., the level of similarity was manipulated within participant).

All children learned the same 16 facts, but the grouping of the facts into 4 sets was different for each child (Additional file 1: Table S4). The per-child grouping was random and was designed to maximize the difference between the low-similarity and the high-similarity sets. We also ensured that similarity was not confounded by problem size—i.e., that the low-similarity sets were not consistently easier due to smaller operands. In fact, the average operand size in the low-similarity sets was slightly *higher* than in the high-similarity sets (Table 1. For the excluded participants, there was a very small difference in problem size, Additional file 1: Table S5). The participants were not told about the similarity manipulation. The experimenters knew about the manipulation but they were not told, for each specific week, whether it was high-similarity or low-similarity.

**Table 1:** Statistical information about the stimuli. Each participant learned the same 16 multiplication facts, which were grouped into 2 low-similarity sets and 2 high-similarity sets (different grouping for each participant)

Participant	Similarity in…		Similarity of the set trained in week 1	Average operands size in…	
	Low-similarity sets	High-similarity sets		Low-similarity sets	High-similarity sets
1	2, 2	11, 11	High	6.75	6.00
2	2, 2	11, 11	Low	6.25	6.50
4	2, 2	11, 11	Low	6.69	6.06
6	1, 1	11, 12	High	6.50	6.25
7	1, 2	11, 11	Low	6.44	6.31
9	0, 1	11, 11	Low	6.44	6.31
11	1, 1	10, 11	High	6.13	6.63
12	2, 2	11, 11	Low	6.44	6.31
13	2, 2	10, 10	High	6.56	6.19
16	2, 2	10, 10	High	6.63	6.13
19	2, 2	10, 10	Low	6.88	5.88
23	1, 1	10, 11	High	6.13	6.63
25	2, 2	12, 12	High	6.44	6.31
27	1, 1	10, 10	Low	6.81	5.94
31	2, 2	11, 11	High	6.56	6.19
33	0, 1	10, 11	Low	6.56	6.19
34	2, 2	11, 11	High	6.00	6.75
Average	1.56	10.76		6.48	6.27

Each set was learned in a separate week (4 weeks overall), in counterbalanced order (either low–high-low–high or high-low–high-low). We made sure that the low-similarity sets were not easier than the high-similarity sets in terms of problem size

#### Computing similarity

There are many possible methods to compute the similarity among facts in a given set. Different methods rely on different theoretical assumptions about several issues, e.g., whether the similarity index should reflect the similarity between digits or between words, whether it should be sensitive to the role of a particular digit/word as an operand or a result, etc. (cf. Appendix of Dotan & Friedmann, 2019). In the absence of a systematic comparison between the different similarity computation methods, we used a simple method that previous studies have shown to be effective (De Visscher & Noël, 2013, 2014a, 2014b; Dotan & Friedmann, 2019). First, the similarity between two facts was defined as the number of digit pairs that appeared in both facts, irrespective of the digits’ position in the fact and their role as operand or result. For example, the facts 8 × 7 = 56 and 8 × 3 = 24 have no common digit pair (only the digit 8 appears in both) so their similarity is 0. The facts 3 × 4 = 12 and 3 × 7 = 21 have 3 common digit pairs (1–2, 2–3, and 1–3) so their similarity is 3. Then, the similarity index for a set of 4 facts was computed by summing the pairwise similarities of all 6 fact pairs in the set. For the multiplication table up to 9 × 9, this similarity index essentially reflects the overlap between the digits of the two facts (operands and response), with no penalty for a single overlapping digit and a nonlinear penalty for additional overlapping digits.

### Procedure

**Fig. 1:**
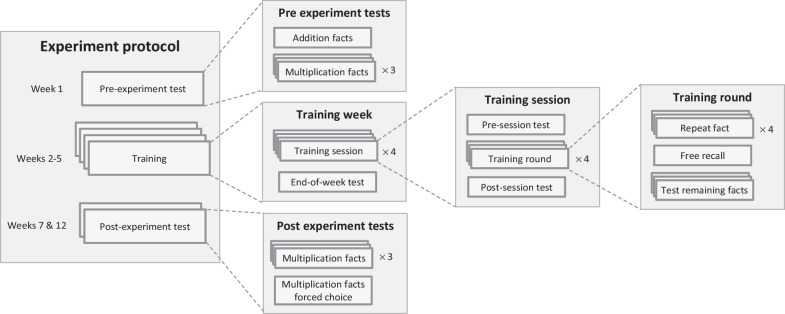
The experiment protocol. The experiment included a pre-experiment testing week (week 1), four training weeks (weeks 2–5), and two post-experiment testing weeks (weeks 7 and 12). Each testing week included 3 sessions, each of which involved testing the child on each multiplication fact as an open question. The post-experiment testing weeks also included a fourth session with a forced-choice test. Each training week included 4 training sessions in which that week’s 4 facts were rehearsed, followed by a session that tested all facts trained in the present and previous weeks. A training session consisted of a pre-session test (each of the week’s 4 facts tested once; a week’s first training session did not include a pre-session test), 4 training rounds, and a post-session test (each fact tested once). In a training round, the child first heard the 4 facts and repeated them, then he was tested on them

The experiment sessions were held during the COVID-19 pandemic period, which prevented face-to-face meetings, so they were done in one-on-one online video meetings with voice over a phone call. To reduce the effects of inter-individual differences, the design was fully within-participant, i.e. all children underwent the same procedure. The experiment started with one week of pre-experiment tests (week 1; Fig. 1). At this time the children did not yet start learning, so obviously, they almost invariably answered “I don’t know”. The pre-experiment test was aimed primarily to verify that the participants had no prior knowledge of multiplication. Four training weeks followed immediately (weeks 2–5). After a delay of one week there was a post-experiment test (week 7), and another post-experiment test after an additional delay of 4 weeks (week 12). The experiment weeks were not aligned with calendar weekdays. To increase the relevance to school-learning situations, we aimed to examine the specific effect of similarity on learning (as opposed to its effect on testing/retrieval, as in Campbell, 1987), so we applied the similarity manipulation during the learning sessions but not during the test sessions. In the test sessions, similar and dissimilar facts were mixed.

#### Training period (weeks 2–5)

Each training week included 5 sessions, held on 5 different days: 4 training sessions (approximately 20 min each) in which that week’s 4 facts were rehearsed, followed—with a gap of at least one day—by a weekly test session. Each training session consisted of a pre-session test, 4 training rounds, and a post-session test.

#### Training rounds

Each training session included 4 training rounds, which were the core of learning. Each round started with the experimenter saying each fact and the participant repeating it. Errors were corrected immediately. The participants then retrieved all facts they remembered (exercise and result), and the experimenter corrected any errors. Finally, the experimenter asked about any fact that the child did not retrieve, with immediate error correction. Whenever an error was corrected, the participant repeated the correct fact (exercise and result) until saying it correctly. The order of presenting the facts was random and was different for each presentation of the 4 facts (counterbalanced within participant).

#### Pre- and post-session tests in the training sessions

These tests were held at the beginning and end of each training session. A week’s first training session did not include a pre-session test, as the child did not yet start learning that week’s facts. Both tests had the same structure: the child was tested once on each of the week’s 4 facts by asking “how much is X times Y?” After the child responded to all 4 facts, errors were corrected: for each fact in which the child erred or did not know the answer, the experimenter said the exercise and the solution, and the child repeated it. If the child repeated incorrectly, the experimenter asked to repeat the fact over and over again, until it was repeated correctly.

#### Weekly test sessions during the training period

In the last session in each week, the child was tested on each of the facts learned so far in the current and previous weeks—i.e., on 4 facts at the end of the first training week, and on all 16 facts at the end of the 4th training week. This weekly test included 2 rounds, each presenting all tested facts in random order (different order in each round), with the limitation that the 4 facts of the current week were the first in each round. No feedback was given except general encouragement. In these weekly tests and in the pre-experiment and post-experiment tests (described below), low-similarity and high-similarity exercises were mixed in the same session.

From these weekly tests, we analyzed only the most reliable responses. To determine how reliable each particular response is in terms of informing about the effect of similarity, two main factors need to be considered. First, in each week the participants were tested on the facts learned in the current week and the preceding weeks, but we analyzed only the responses to the current week’s facts, to avoid confounding with the time elapsed since learning. Second, in each weekly test session, the participants were tested on each fact twice, in two rounds. From these two rounds, we deemed the first round as more reliable, because presumably, this round reflects the participant’s long-term knowledge better than the second round (below, we shall see results indicating that this was indeed the case). The responses in the second round may be confounded by local effects—e.g., the participants may simply repeat their response from the first round. Thus, throughout the results section, the weekly test analyses refer only to the first-round responses, unless explicitly said otherwise.

#### Pre-experiment testing (week 1)

Week 1 included 4 testing sessions, held on 4 separate days. In the first session, the child was tested on 10 single-digit additions and 7 subtractions with a single-digit subtrahend and result. The results of these addition/subtraction tests are reported in Additional file 1: Tables S5 and S6. In each of the next 3 sessions, the child was tested on 43 multiplication facts (each fact once per session, i.e. 3 times during the week): all operand pairs between 2 × 2 and 9 × 9 (36 facts, the larger operand appeared second), and 7 facts with 0 or 1 as operands. The question was “how much is X times Y?” No feedback was given except general encouragement.

#### Post-experiment testing (weeks 7 and 12)

Each of these two weeks included 4 testing sessions, held on 4 different days. The first 3 sessions tested the multiplication facts knowledge like in the pre-experiment tests. The 4th session was a two-alternative forced-choice test. The distracter for a fact was the result of another fact from the same 4-fact set. In week 7, the forced-choice test included a single round with 16 questions—each trained fact appeared once. In week 12, the test included two rounds, each round asking once about each of the 16 trained facts. Untrained facts were not presented in the forced-choice tests. No feedback was given except general encouragement.

### Statistical analyses

For each fact, we defined two measures reflecting its similarity to the 3 other facts in the same set. *Numeric Similarity* is the specific fact’s average similarity to the 3 other facts in the set, and *Similarity Level* is the set’s classification as low-similarity or high-similarity. The grouping of facts into sets was different for each child, so these parameters were computed for each child.

To examine the effect of similarity on the participants’ accuracy, we submitted the per-fact accuracy (correct/incorrect) to a logistic linear mixed model (LLMM) with the Participant and Fact as random factors. The critical within-participant factor was Similarity, the specific similarity predictor being either Numeric Similarity (numeric factor) or Similarity Level. These LLMMs also included two within-participant covariates accounting for the problem size: the average and product of the operands (but in all the critical analyses, the results were essentially the same when removing the two problem-size factors, and also when additionally removing the Fact random factor). Other LLMM configurations are detailed below.

In the few cases wherein an LLMM reached a singular fit, which may result from over-fitting, or did not converge, we first tried z-scoring each predictor. If that did not help, we removed the Fact random factor (similar results were obtained when keeping this factor). To examine the significance of a particular factor or interaction, we used a log-likelihood ratio test and compared the full LLMM to a model in which that factor/interaction was removed. For these comparisons, we report the test statistic 2(LL_1_− LL_0_), which follows a *χ*^2^ distribution (LL_0_ and LL_1_ denote the log-likelihoods of the reduced model and the full model), and the corresponding *p*-value.

## Results

### Learning dissimilar facts is easier

**Fig. 2:**
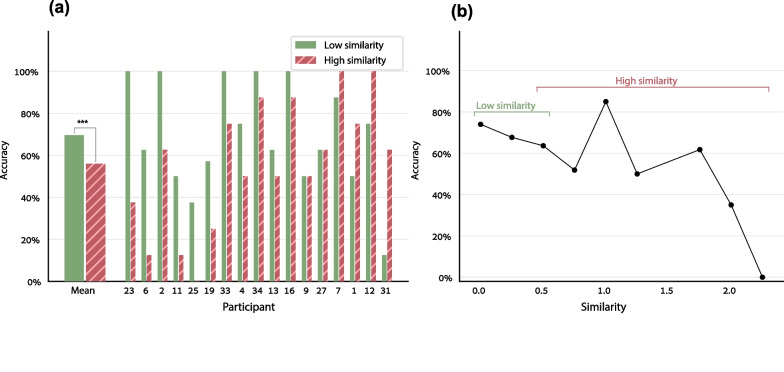
Results of the end-of-week tests—better learning of dissimilar facts than similar ones. **a** The children were more accurate in the low-similarity weeks than in the high-similarity weeks. The thinner bars show the individual results of each participant. **b** Accuracy decreased as a function of the fact’s average similarity to the 3 other facts learned in the same week, even within the low-similarity sets and the high-similarity sets, suggesting that the fact-specific similarity is important

In the end-of-week tests, as predicted, accuracy in the low-similarity weeks was higher than in the weeks with similar facts by 13.7% (Fig. 2a), i.e., by a factor of 1.25. To examine the similarity effect statistically, we used the logistic linear mixed model described in Methods. The dependent variable was the per-fact accuracy, the participant and fact were random factors, and there were 3 within-subject factors: similarity (either Numeric Similarity or Similarity Level, in two separate analyses), and the two problem-size factors as covariates (sum and product of the operands). The effect of similarity was significant (with Similarity Level factor: *χ*^2^(1) = 7.34, *p* = 0.007, odds ratio = 0.41, Fig. 2a; with Numeric Similarity factor: *χ*^2^(1) = 10.47, *p* = 0.001, odds ratio = 0.43, Fig. 2b; detailed LLMM results in Additional file 1: Table S10a, b). Because the number of participants was not large, we verified that the effect of similarity was significant also when comparing the high- and low-similarity sets using statistical tools simpler than a linear mixed model—paired *t* test (*t*(16) = 1.90, one-tailed *p* = 0.04, Cohen’s *d* = 0.48) and Wilcoxon Signed Rank test (*W* = 91, one-tailed *p* = 0.04). An effect of similarity was found also among the excluded participants (specifically, for the 6 participants whose amount of data sufficed to test a similarity effect; Additional file 1: Figure S2). Thus, as predicted, it was harder for the children to learn facts that were similar to each other, and it was easier for them to learn dissimilar facts.

The results above refer to the participants’ first round of responses in each weekly test session, as we considered the responses in this round to reflect the participants’ long-term knowledge more reliably than the responses in the second testing round. Still, the similarity effect was observed also in the second round (same LLMM, Numeric Similarity effect: *χ*^2^(1) = 6.72, *p* = 0.01, odds ratio = 0.49; but the effect was not significant when using the Similarity Level factor, *χ*^2^(1) = 2.48, *p* = 0.12; Additional file 1: Figure S3, Table S11). The similarity effect in the second-round responses was smaller than in the first-round responses, in line with our assumption that the first round is a better reflection of the learning and the similarity effect.

Critically, the similarity effect originated in the learning that occurred during the experiment, and cannot be attributed to pre-existing knowledge. In the pre-experiment tests on multiplication facts (in week 1), the average accuracy on the to-be-trained facts was virtually zero, leaving no room for a similarity effect: 2.7% in the low-similarity sets and 2.5% in the high-similarity sets. The 0.2% difference between the low- and high-similarity sets was not significant (a logistic linear mixed model did not converge, so we used paired *t* test: *t*(16) = 0.32, one-tailed *p* = 0.37).

Contrary to findings with older children (De Visscher & Noël, 2014a), the participant’s individual sensitivity to similarity (hereby StS, Fig. 2a) did not predict their overall accuracy. To examine this, we entered the per-participant overall accuracy as the dependent variable in a multiple linear regression. The critical predictor was the participant’s StS, defined as Δaccuracy between low- and high-similarity sets (Δaccuracy > 0 denotes higher sensitivity). A second predictor controlled for the fact that each child learned the multiplication facts in a different grouping, with different specific levels of similarity. This predictor was defined as $\Delta{\text{similarity}}={\text{Sim}}_{H}-{\text{Sim}}_{L}$, with Sim_H_ and Sim_L_ denoting the participant’s average within-set numeric similarity of the two high-similarity sets and the two low-similarity sets, correspondingly. If the participant’s StS affects the overall accuracy, the StS predictor should have a significant negative effect. This was not the case: neither predictor had a significant effect (StS: *b* = − 0.55, one-tailed *p* = 0.21; Δsimilarity: *b* = − 0.12, one-tailed *p* = 0.30). Still, crucially, high sensitivity to similarity predicted low performance in the high-similarity sets: in a similar regression, in which the dependent variable was accuracy in the high-similarity sets, the StS predictor had a significant negative effect (*b* = − 0.61, one-tailed *p* = 0.006), with no effect of Δsimilarity (*b* = − 0.57, one-tailed *p* = 0.20). Such an effect of StS was not found when the dependent variable was accuracy in the low-similarity sets (StS: *b* = 0.39, i.e., opposite to the predicted direction; Δsimilarity: *b* = − 0.57, one-tailed *p* = 0.20). In sum, sensitivity to similarity disrupted learning specifically in high-similarity conditions.

The findings above clearly show that it is easier to learn dissimilar facts than similar ones. In the subsequent sections, we examine why this is so.

### The similarity effect arises specifically from the grouping of facts in the training sessions

What is the origin of the similarity effect? Our assumption, presented in the Introduction, was that the similarity effect originated in the specific grouping of facts into weekly sets during the learning time. In particular, we assumed that high similarity between the facts in a given week (hereby, “within-set similarity”) would disrupt learning. Correspondingly, the similarity index we defined captures the similarity between each fact and the 3 other facts in the same week.

An alternative view is that learning is disrupted by the similarity between the current week’s facts and the facts learned in previous weeks. This resembles the idea proposed by de Visscher and Noël (2014b): they assumed that the multiplication facts are learned in a certain order, and that learning is modulated by the similarity between each fact and all earlier facts in this “learning list”. A similarity index reflecting this alternative view should capture the similarity between each fact and all the facts in the previous weeks.

To arbitrate between these two alternatives, we ran an LLMM on the weekly test data with accuracy as the dependent variable, the participant as a random factor, and with four within-participant factors. Two similarity factors represented the two hypotheses above: *Within-set Numeric Similarity*, defined as in the previous section, and *Similarity versus Previous Weeks*: the average pairwise similarity of each fact in the current week versus each fact learned in previous weeks. For facts learned in the first week, for which there are no preceding weeks, this factor was defined as 0. Additionally, the LLMM included the two problem-size factors—the sum and product of the two operands. This analysis showed a significant effect of Within-set Similarity (Table 2): for each additional point in the within-set similarity index, the odds of responding correctly in the end-of-week test decreased by a factor of 0.44. In contrast, there was no significant effect of Similarity versus Previous Weeks. Thus, the critical factor that affects learning is the similarity of a fact versus the other facts learned in the same week.

**Table 2:** The results of the logistic linear mixed model that examined whether accuracy in the end-of-week tests was affected by the similarity between the facts in each week (within-set) or by the similarity of each set of facts versus the previously learned facts

Factor	Odds ratio	95% CI	Significance
Intercept	41.14	0.04–46,680	
Within-set numeric similarity	0.44	0.25–0.77	*χ*^2^ = 9.53, *p* = 0.002
Similarity versus previous weeks	1.16	0.55–2.43	*χ*^2^ = 0.14, *p* = 0.71
Sum of operands	0.77	0.29–2.0	
Product of operands	1.03	0.89–1.18	
*Random effects*			
σ^2^ within participant and exercise	3.29		
σ^2^ between participants	1.47		
σ^2^ between exercises	0.35		

A significant effect was found only for the within-set similarity

The effect of the per-fact Numeric Similarity could be observed to some extent even when controlling for the per-set Similarity Level. We showed this using LLMM with 4 predictors –Numeric Similarity, Similarity Level, and the sum and product of the two operands. The dependent variable was the per-fact accuracy, and the Fact and Participant were random factors. Numeric Similarity had a marginally significant effect with *χ*^2^(1) = 3.14, *p* = 0.08, odds ratio = 0.8 (Additional file 1: Table S12), suggesting that it is the specific similarity level that counts. Figure 2b shows how the set’s specific similarity level affected performance on top of its dichotomous classification as a low-similarity or a high-similarity set.

### The similarity effect arises from long-term memory

Once learned, the multiplication facts are stored in long-term memory. Similarity clearly affected these long-term memory representations, as its effect was observed in the end-of-week tests, which were conducted two days after learning has ended. But how did this effect take place?

One interpretation of the similarity effect could be actually in terms of working memory (WM): if two items have some identical features, this impairs their representations in WM due to interference (Baddeley, 1966a; Bunting, 2006; Oberauer & Lange, 2008). It could be that in high-similarity weeks, the children created poor WM representations of the facts, and as a result, they had less opportunity to construct appropriate long-term memory (LTM) representations. According to this view, the similarity effect did not arise from LTM per se, but “leaked” from WM. An alternative view is that similarity affected LTM directly. It is also possible that both LTM and WM give rise to similarity effects. Indeed, similarity can affect both WM and LTM (Baddeley, 1966b).

Two aspects of our data may arbitrate between these two views: (1) *Performance at the beginning versus the end of each training session*. If the similarity effect originates in WM, it should be observed in the post-session tests, when WM representations are stronger, but should be weaker or completely absent in the pre-session tests. In contrast, if the similarity effect originates in LTM, it should be observed in the pre-session tests: these tests are affected by long-term representations, and less affected by the momentary WM activation by the current training session, which has not yet started. (2) *Day-by-day progress*. If the similarity effect originates in WM, it should be observed from the very first session, because each session recruits the WM. In contrast, if the similarity effect originates in LTM, its effect may kick in only later, after a few days, when sufficiently strong LTM representations have been created.

To examine these predictions, we used multiple LLMMs and analyzed separately the pre-session test and the post-session test of each day. The dependent variable was accuracy, the participant and fact were random factors, and there were 3 within-participant factors: Numeric Similarity, and the sum and product of the operands.

**Fig. 3:**
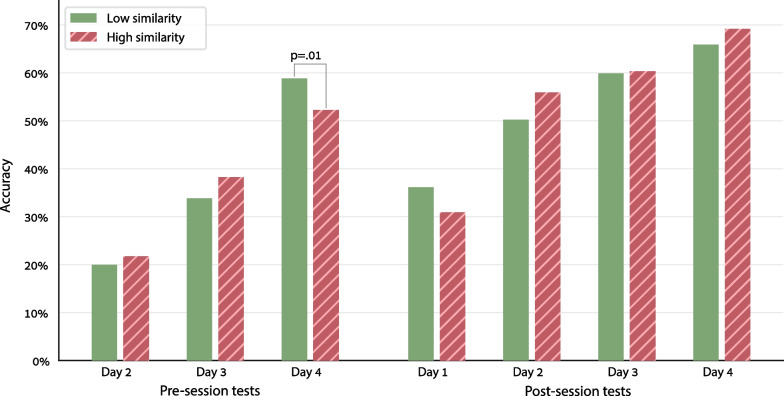
Accuracy in the short tests conducted at the beginning and end of each training session. The overall performance improves from day to day. Critically, a similarity effect was observed only in the pre-session test of the last training day—the test that was most sensitive to long-term memory representations. In all other pre- and post-session tests, the similarity effect was not significant

The results (Fig. 3) confirmed the predictions of the LTM-originated-interference view. A significant effect of similarity was observed in the pre-session test of the 4th training day (*χ*^2^ = 6.5, *p* = 0.01, odds ratio = 0.83, Additional file 1: Table S13), but not in the pre-session tests of days 2 and 3 (both *p* > 0.90). The similarity effect in day 4 was stronger than in the pre-session test of the preceding days: a similar LLMM on all 3 days, with Day (#4 vs. 2+3) and Day  × Similarity interaction as additional factors, showed a significant interaction (*χ*^2^ = 4.31, *p* = 0.04, odds ratio = 0.68, Additional file 1: Table S14). Thus, the predictions of the LTM-originated-similarity hypothesis were fully corroborated: there was a significant effect of similarity in the pre-session test, and this effect was not observed on the first days of training but appeared only on the last day. In contrast, the prediction of the WM-originated-similarity hypothesis was refuted: no significant effect of similarity was found in any of the post-session tests (all *p* > 0.11).

**Fig. 4:**
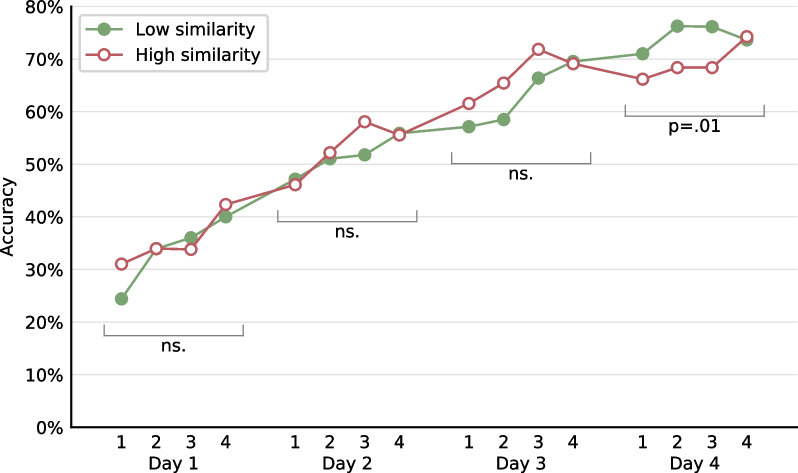
The effect of similarity on accuracy in each response attempt during the training rounds. The improvement was nearly monotonous from day to day, with a significant effect of similarity only on the last training day—the day most sensitive to long-term memory representations

The finding of a similarity effect only on day 4 was replicated also when examining the participants’ responses during the training rounds (Fig. 4). Accuracy increased with training, and critically, the effect of similarity emerged only on the last training day, again supporting the idea that similarity affects LTM. We ran an LLMM on the accuracy in each response attempt, with the participant and fact as random factors, and with several within-participant factors: Numeric Similarity, the sum and product of the operands (to account for problem size), the training round (1–4, numeric factor), and the serial position of the item in the specific round (1–4, numeric factor). Again, the effect of similarity was significant in day 4 (*χ*^2^(1) = 6.03, *p* = 0.01, odds ratio = 0.70, Additional file 1: Table S15) but not in the preceding days (all *p* > 0.65).

Altogether, these findings indicate that similarity affected the long-term memory representations directly, without the mediation of working memory processes. Still, in the next section, we will see evidence suggesting that working memory processes may be involved in some way in creating the effect of similarity.

### Low-similarity training is beneficial also in the long run

#### An effect of similarity after several weeks

**Fig. 5:**
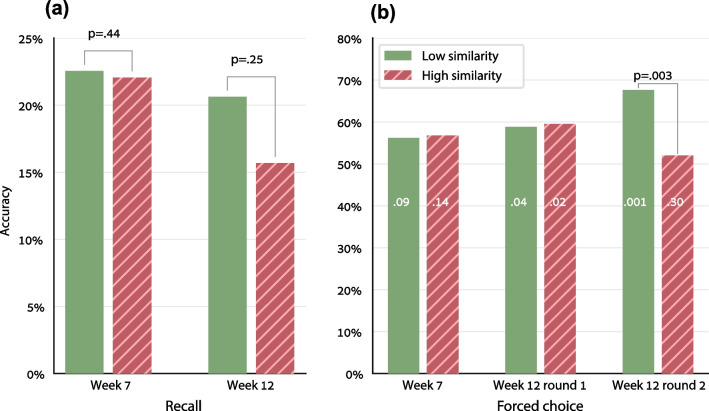
The effect of similarity on accuracy in the post-experiment tests—the recall tests (average of sessions #1–3 in each week) and the two-alternative forced-choice tests (session #4 in each week). Only the trained facts are shown here. The *p* values above the columns are the effect of the Numeric Similarity factor in the LLMM described in the text. The *p* values inside the forced-choice columns refer to comparing the per-participant mean accuracies to chance level (paired t-test against 50%, one-tailed *p*)

Unsurprisingly given the relatively small amount of training, accuracy in the post-experiment free recall tests was not high—about 20% (Fig. 5a). Still, the post-experiment forced-choice accuracy in week 12 was higher than chance (Fig. 5b)—i.e., although we provided relatively little training, the children had memory traces of the multiplication table several weeks after training has ended. The critical question is whether manipulating similarity during training affected the long-term representations. Figure 5b indicates that it did: in the second round of the forced-choice test in week 12, performance was good in the low-similarity items (68% success; comparing the per-participant success rate to the 50% chance level, paired *t*(16) = 3.68, one-tailed *p* = 0.001, Cohen’s *d* = 1.30) but not in the high-similarity items (*p* = 0.31). The difference between low- and high-similarity items was significant. We examined this using an LLMM on the post-test accuracy of the week 12 s round of forced-choice, with Numeric Similarity and the operands’ sum and product as within-subject factors, and with the participant as a random factor (the fact random factor was not added because this model did not converge). The Similarity effect was significant (*χ*^2^(1) = 9.03, *p* = 0.003, odds ratio = 0.67, Additional file 1: Table S16). Such similarity effect was not found in the previous forced-choice tests—the one in week 7 and the first forced-choice test in week 12 (numerically, the performance was even higher for high-similarity facts, as can be seen in Fig. 5b, but this effect was not significant, *p* > 0.22). Importantly, the *p *value obtained for the similarity effect in the last forced-choice test (*p* = 0.003) survives a Bonferroni correction for the multiple comparisons in 5 post-experiment tests.

We acknowledge that while our findings in the previous sections showed a clear and robust effect of similarity on long-term memory two days after learning has ended (in the end-of-week tests), the support for a weeks-long effect of similarity is not as strong, as the long-term effect was observed only in the very last post-experiment tests. Still, note that the specific pattern observed here—an effect of similarity in week 12 but not in week 7—does not appear to be random, because it replicates a previous study that used the same paradigm (Dotan & Friedmann, 2019).

#### The weeks-long similarity is not an experimental artifact

We ruled out an alternative, short-term memory interpretation of the week 12 similarity effect. This interpretation emphasizes that the week 12 forced-choice test included two rounds, with different distracters in each round, and the children may have relied on this information. For example, if 7 × 6 = 42 was presented with distracter 35 and then with distracter 63, in the second round they could deduce that the correct answer was 42—the only alternative that appeared in both rounds. Critically, the ability to remember the exercise and the distracters could be affected by the within-set similarity and mediated by short-term memory.

To examine this “strategic guessing” interpretation, we recruited another group of 20 first-grade children (mean age = 7;1, *SD* = 0;3) who performed only the two-round forced-choice test, with no preceding learning. Three children were excluded for stereotypical answers (consistently choosing the second alternative, Additional file 1: Table S3). The strategic-guessing view predicts that in this experiment too, the performance in the second round will be (1) higher than chance, (2) higher than in the first round, and (3) affected by similarity. All three predictions were refuted. First, accuracy in the second round was low (47.0%)—less than the 50% chance level. Second, accuracy in the second round was lower, not higher, than in the first round (53.8%). Third, accuracy in the second round was unaffected by similarity—it was almost the same in the low-similarity sets (48.7%) and the high-similarity sets (45.3%). The difference between low- and high-similarity sets was not significant (LLMM on accuracy with the participant as a random factor, and Numeric Similarity and the sum and product of the operands as within-participant factors, the effect of similarity was not significant: *χ*^2^(1) = 0.03, *p* = 0.87; similar results when using Similarity Level instead of Numeric Similarity, *χ*^2^(1) = 0.38, *p* = 0.54). These results refute the strategic-guessing interpretation and support the idea that the similarity effect in week 12 indeed originated in long-term memory.

## Discussion

### Low similarity facilitates memorization

This study examined whether young children, who do not yet know the multiplication facts, learn them better when dissimilar rather than similar facts are learned together. The children memorized sets of facts with low or high within-set similarity, each set during one week. The results were clear: by the end of a learning week, accuracy in the low-similarity sets was higher than in the high-similarity by a factor of 1.25. This difference cannot be attributed to fact-specific characteristics such as problem size, because the facts were grouped differently for each child, and because our analyses controlled for the problem size. Thus, the similarity effect did not originate in an intrinsic property of particular facts, but had to do with how we grouped the facts during the learning sessions. Because similarity was manipulated, we can conclude causality: low similarity not just correlates with multiplication fact knowledge, it improves knowledge.

The best predictor of performance was the fact’s precise similarity to the 3 other facts learned in the same week, not its similarity to the facts learned in the previous weeks. We conclude that the main source for similarity-induced interference is from the simultaneous learning of similar facts, not from learning a new fact that is similar to an already-learned one. This conclusion reaffirms those of De Visscher and Noël (2014b): they too showed that a similarity measure should consider the order of learning the arithmetic facts. It also extends De Visscher & Noël’s conclusions in two critical respects. First, whereas their similarity measure relied on a fixed learning order, using assumptions based on the school curriculum, here we knew the precise learning order for each child. Second, here we could arbitrate between a putative effect of interference from previously learned facts and an effect of interference from simultaneously learned facts, and show that the latter is the critical one.

Our results join a growing number of studies showing that similarity disrupts memorization of arithmetic facts (Campbell, 1987; De Visscher & Noël, 2013, 2014a, 2014b; De Visscher et al., 2018; Katzoff et al., 2020; Polspoel et al., 2019). The exact definition of similarity, however, is still an open issue: we do not yet know what precisely makes two facts similar to each other (cf. Appendix of Dotan & Friedmann, 2019).

### The specific memory mechanisms underlying the similarity effect

#### Similarity affects long-term memory

Our data indicates that the similarity between facts specifically affected long-term memory representations. First, in the day-by-day learning curve, the effect of similarity was not observed right from the start, as should have been the case if similarity had a momentary effect on short-term memory, but emerged only on the last training day, after long-term memory representations were sufficiently established. Second, the effect was observed in the pre-session test, which presumably reflects long-term memory representations, and not in the post-session test, which presumably reflects short-term effects from the just-ended session. Third, a strong effect of similarity was observed in the end-of-week tests, which were held 2 days after the last training session. Fourth, an effect of similarity, albeit smaller, was observed even in week 12 of the study, 7 weeks after training has ended.

#### The weeks-long similarity effect

The effect of similarity in the post-experiment tests is particularly interesting because it shows that manipulating similarity during learning has a long-lasting effect. This weeks-long similarity effect was observed in the last post-experiment test, but not in the preceding post-experiment tests. There are at least two aspects of the discrepancy between the different post-experiment tests, and they may have different origins. The first aspect is that the similarity effect was not observed a few days after the end of the training period, but it was observed several weeks later. This discrepancy is not surprising because it replicates a single-case study that used a design similar to ours (Dotan & Friedmann, 2019): in this study too, the participant did not show a similarity effect in the post-experiment test administered shortly after the 4-week training period, but she did show a similarity effect in a test administered 2 months later. One interpretation of this discrepancy could be that long-term memory processes continue operating even weeks after the learning sessions have ended, and the efficiency of these processes depends on how the facts are encoded, which in turn depends on the learning-time similarity. Another possibility is that when the test was run only a few days after the end of the learning period, certain confounding factors had a stronger effect—e.g., the time elapsed since learning a particular fact. The variance of this factor shortly after the training period was higher than its variance 5 weeks later.

A second aspect of the discrepancy between the post-experiment tests is that even in the last testing session, in which the effect was observed, it was observed in the second round of testing but not in the first round. A possible explanation is that although the similarity effect originates in long-term memory, as shown above, its impact on recall can occur only after increasing the facts’ activation level. In the week 12 forced-choice tests, this “warmup” was presumably triggered by the experiment protocol—the first testing round in this session served as warmup to the second round, resulting in a similarity effect in the second round. In contrast, there was no warmup to the first round, hence no similarity effect. There was no warmup also in the free-recall post-experiment tests, in which each fact was presented only once. In the weekly tests during the training period, the experimental protocol did not trigger such memory warmup, however, such warmup may have occurred implicitly. For example, because the participants still remembered the facts relatively well during these weekly tests, they may have activated the facts to a sufficient level even before being asked about them. Future studies may examine how long- and short-term effects interact to create the similarity effects (Katzoff et al., 2020). Another question is which of the forced-choice tests best reflects the pedagogical reality—the first testing round or the second. Arguments could be made in either direction; but at least one argument supports the idea that the second testing round mimics real school scenarios better than the first testing round: at school, more often than not, children are not required to answer a single multiplication question, but to solve several mathematical problems one after another. The child would therefore not encounter each fact once, as is the case in the first testing round, but several times in a row, as is the second testing round.

We acknowledge that while the finding of a weeks-long similarity effect was significant (even after correcting for the multiple comparisons), it was not as robust as the findings from the end-of-week tests. Replicating the weeks-long similarity effect, preferably with more intensive learning than we provided here, may be the goal for future studies. An interesting question is whether such studies would also replicate the two aspects of discrepancy between the different post-experiment tests—the finding of a similarity effect weeks after the experiment but not earlier, and the memory warmup effect.

#### Does similarity affect conceptual learning?

Another question is whether, although our training program was aimed specifically to improve rote memorization of the multiplication facts, it also helped the children gain implicit knowledge about the meaning of multiplication or the rules underlying the multiplication table. If such knowledge was indeed gained, and more so for the low-similarity sets, it might provide another explanation for the similarity effect. Our study was not designed to examine conceptual knowledge, but we did not observe any overt evidence for such knowledge. Visual inspection of our stimuli actually seems to contradict the conceptual-similarity-effect account: some regularities appear to be more transparent in the high-similarity sets, e.g., these sets often included a sequence of same-operand exercises such as 6 × 7, 6 × 8, 6 × 9. Future studies may examine further whether low-similarity training has an impact (either positive or negative) on conceptual learning of multiplication.

### Individual differences in sensitivity to similarity

Different children had different degrees of sensitivity to similarity-induced interference: some children benefited from low similarity more than others, and few children even showed an opposite trend. An open question is whether this variance reflects random noise, or whether there is a genuine difference between the children who gained from low similarity and those who did not.

The per-child degree of sensitivity to similarity-induced interference (StS) correlated with the performance in the high-similarity sets. Previous studies too found that high StS predicted poor knowledge of arithmetic facts (De Visscher & Noël, 2014a, 2014b). Interestingly, the precise kind of correlation between StS and performance was different in De Visscher and Noël’s studies and in ours. In our study, the StS correlated specifically with the performance in high-similarity sets, i.e., the individual differences were reflected in the more-demanding condition (sets with high similarity). In contrast, in De Visscher and Noël (2014a), who examined 4th-grade children, StS correlated with the overall performance. A possible interpretation of this discrepancy is as follows: when children only start learning the multiplication table, the individual differences in StS affect only the difficult situations. As the children grow up, the gap between them is not closed but widened. Note, however, that conclusions from these correlations should be taken with caution because our sample size was not large enough for reliable between-participant analyses.

In extreme cases, an abnormal degree of sensitivity to the similarity-induced interference (“hypersensitivity to interference”) may cause abnormally poor knowledge of arithmetic facts—dyscalculia (De Visscher & Noël, 2013; Dotan & Friedmann, 2019). The training program we used here seems to be effective also for individuals with hypersensitivity to interference (Dotan & Friedmann, 2019), perhaps even more than for individuals without this disorder. Indeed, the similarity effect size observed in Dotan and Friedmann (2019), who used the same method with a woman with hypersensitivity to interference, was considerably larger than the effect size observed here. Still, our findings show that low-similarity training is effective also for children with normal levels of StS.

### Generalizability of the conclusions

Several factors indicate that our findings are reliable even if the sample size was not large (17 participants). First, we examined the performance at multiple time points: before each training session, at the end of each training session, the individual response attempts during training, at the end of each training week, and after the experiment had ended. Similarity demonstrably affected performance in several of these time points—in the response attempts during the training session, at the beginning of the training session, in the end-of-week tests, and in the post-experiment test. Second, the specific time points in which a similarity effect was found were predicted by theoretical considerations (e.g., the similarity-in-LTM view predicted the difference between the pre-session and post-session tests) and by previous studies (e.g., the pattern of similarity effects in the post-experiment tests replicated Dotan & Friedmann, 2019). Third, an effect of similarity was found also among the excluded participants. Fourth, the impact of similarity was observed in several different types of analyses: in the accuracy of the weekly and post-experiment tests, in the day-to-day progress patterns, and in the comparison between pre-session and post-session tests. Last, the effect of similarity was observed with high significance levels and large effect sizes. For all these reasons, the results cannot be deemed unreliable due to a small dataset size or a statistical bias in a particular analysis.

The participant dropout rate was 50%, but critically, this too does not hamper the reliability of our conclusions about the effect of similarity, because the participant exclusion criteria were completely orthogonal to similarity. No child was excluded on grounds related to similarity. Except for one child, who decided to quit for an unknown reason, children were excluded because they had pre-existing knowledge of multiplication (3 children) or because they were generally inattentive or non-cooperative (14 children). Furthermore, an effect of similarity was found even among the excluded participants.

While the results are reliable, an open question is to what extent they can be generalized to a wider population. We see two main limitations to generalizability. One limitation is the recruitment method: we did not recruit whole classes systematically, but individual participants via social networks on a voluntary basis. This recruitment method may potentially be biased. The second limitation is the participant dropout. Our main exclusion criterion was inattentiveness, and we should consider the possibility that the excluded children’s inattentiveness was not arbitrary but resulted from low attentional abilities. An extreme hypothesis, which we cannot corroborate or refute, is that *most* children with lower attentional abilities were excluded from the study. This would mean that our findings may be true for typically developing children but not for those with low attentional abilities or with attention disorders. Note that the assumption that attention is related to similarity and memorization is theoretically justified: a main aspect of attention, in particular executive attention, is the ability to cope with cognitive conflicts and interference; and executive attention is tightly related to (or even fully overlaps) working memory abilities (Engle, 2002; Oberauer, 2020; Petersen & Posner, 2012). However, critically, note also that the straightforward prediction from these attention-similarity relations is not that the effect of similarity would be lower for children with low attentional abilities than in our sample, but that it would actually be higher for these children. Somewhat in line with this idea, studies that examined how similarity affects multiplication-table-learning in other populations with difficulties—specifically, mathematical or memory difficulties—showed a strong effect of similarity (De Visscher & Noël, 2013, 2014a; Dotan & Friedmann, 2019).

### Pedagogical implications

Our study showed that when a child needs to memorize randomly grouped arithmetic facts, learning in a low-similarity context is superior to a high-similarity context. From a pedagogical perspective, an important question is whether low-similarity training outperforms also the typical schooling method—learning the multiplication table by columns (the times-3 table, then the times-4 table, etc.). Our data cannot answer this question. An interesting hypothesis is that both low-similarity learning and by-column learning are good methods, yet each method is best suited for learning a slightly different aspect of multiplication. By-column learning may be superior for conceptual learning of the multiplication table—e.g., to understand the meaning of multiplication and its relation with addition, and to learn calculation-based strategies (e.g., if you forgot how much 7 × 6 is, you can compute it as 7 × 5 + 7). Low-similarity training may be superior for rote memorization, because the column-by-column organization tends to group similar facts. If this is indeed the case, a good method to teach multiplication may be to start with column-by-column teaching in school, then switch to low-similarity training for rehearsal in school, homework, computer games, etc.

The interaction between rote memorization and conceptual learning of the multiplication table could be even more complex. In the present study, to focus on the memorization aspect rather than on conceptual learning, we taught the multiplication facts as arbitrary sequences of numbers, without explaining what they mean. While this decision seems methodologically justified, given that adults generally solve arithmetic facts using a retrieval-from-memory strategy (Campbell & Beech, 2014), it is still possible that the cognitive representation of the facts would be different if rote-learning is accompanied by understanding the mathematical semantics of each fact. Follow-up studies may examine the effect of similarity on learning the multiplication table in more ecological settings, i.e., in a classroom and when learning emphasizes both rote memorization and mathematical semantics.

Low-similarity training was tested here for multiplication, but it may be effective also to memorize addition facts, which can be retrieved from verbal memory too (Campbell, 1995; even if additions can be solved by other strategies too, Dehaene et al., 2003). The method may also be effective to memorize other types of information that may be subject to similarity-induced interference—for example, formulas in algebra (${(a+b)}^{2}={a}^{2}+{b}^{2}+2ab$), calculus ($\frac{d{x}^{n}}{dx}=n{x}^{n-1}$), or geometry (${\text{triangle surface}}=\frac{{\text{base}}\times {\text{height}}}{2}$), and even for rote learning of non-mathematical content. This is not unlikely, given that the human memory is known to be affected by similarity and interference in a wide range of tasks, involving different memory processes and different types of content (Baddeley, 1966a; Farrell & Lewandowsky, 2003; Nelson et al., 1974; Oberauer et al., 2012; Pajak et al., 2016; Smith et al., 2021; Sosic-Vasic et al., 2018).

## Conclusion

This study showed that the ability to learn the multiplication table is affected by the similarity between facts, and that this similarity effect arises from long-term memory processes. We further showed that a simple manipulation—a careful grouping of specific arithmetic facts into lessons, based on low similarity to each other—may help to memorize the multiplication table. This method is quite different from the existing school practices, so we hope it may be used to improve how elementary schools teach the multiplication table—an excruciating challenge for many children. We hypothesize that such improvement will not take the form of abandoning the existing teaching methods, but will combine them with low-similarity training.

Our study leaves several open questions. Most importantly, it is still not fully clear which precise memory processes are responsible for the similarity effect in arithmetic fact learning; what precisely makes two facts similar to each other, and how similarity should be measured; and whether and how rote memorization and conceptual learning interact when learning arithmetic facts. Future investigations of these questions may lead to a better understanding of how our brain learns mathematical facts, and what math teaching in elementary school should look like.

## Significance statement

Mastering the multiplication table is one of the foundations of arithmetic fluency and an important aspect of the elementary school curriculum. Learning the multiplication table is also hard: many students spend a tremendous effort learning it, some never succeed, and even as adults many still remember this as an agonizing experience. Several pedagogical programs focus on understanding the concepts and regularities underlying the multiplication table. Here, we focused on a complementary aspect of learning the multiplication table: the rote memorization of individual multiplication facts. We capitalized on a well-known cognitive phenomenon—that human memory is sensitive to the interference arising from the similarity between the items being memorized. This may disrupt rote memorization of the multiplication facts because they are highly similar to each other. As a possible solution to this difficulty, we examined whether multiplication fact learning by young children can be made more efficient by reducing the inter-fact similarity. Indeed, when we taught only relatively dissimilar facts in each given lesson, learning was better than in a control condition in which similar facts were taught in conjunction. We propose that educational programs for teaching the multiplication table, at least those programs that focus on the memorization aspect, should consider the similarity between facts as a critical factor.

## Supplementary Information

**Additional file 1:** Supplementary material.

## Data Availability

The datasets generated and/or analysed during the current study, and the analysis scripts, are available in the OSF repository, http://osf.io/pqtjy.
