# Supplementary material for: Elementary math in elementary school: the effect of interference on learning the multiplication table
Source: Cogn Res Princ Implic. 2022 Dec 2;7:101. doi: 10.1186/s41235-022-00451-0 (PMC9716515; doi:10.1186/s41235-022-00451-0)
Supplement: Supplementary file 1 — Additional file 1: Supplementary material. [file 41235_2022_451_MOESM1_ESM.pdf]

Supplementary material for

Elementary math in elementary school: The effect of interference on learning the multiplication table

Dror Dotan and Sharon Zviran-Ginat

1. Detailed stimulus information

Table S1. The sets of facts trained in each week for each participant. Grey background indicates high-similarity sets. The stimuli are also provided in CSV format.

Participant	Week 1	Week 2	Week 3	Week 4
1	4×7=28, 8×9=72<br>4×6=24, 6×7=42	6×8=48, 6×9=54<br>5×7=35, 3×9=27	3×8=24, 4×9=36<br>3×6=18, 4×8=32	7×8=56, 7×9=63<br>3×7=21, 5×9=45
2	4×7=28, 6×9=54<br>4×6=24, 5×7=35	4×9=36, 8×9=72<br>3×9=27, 7×9=63	6×7=42, 5×9=45<br>3×7=21, 8×7=56	3×8=24, 6×8=48<br>3×6=18, 4×8=32
4	4×7=28, 5×9=45<br>4×6=24, 7×8=56	3×9=27, 7×9=63<br>3×7=21, 4×9=36	6×7=42, 8×9=72<br>5×7=35, 6×9=54	3×8=24, 6×8=48<br>3×6=18, 4×8=32
6	4×8=32, 4×9=36<br>3×8=24, 6×8=48	7×8=56, 7×9=63<br>4×6=24, 5×9=45	4×7=28, 8×9=72<br>3×7=21, 3×9=27	5×7=35, 6×9=54<br>3×6=18, 6×7=42
7	5×7=35, 6×9=54<br>3×6=18, 3×9=27	4×8=32, 4×9=36<br>3×8=24, 6×8=48	7×8=56, 7×9=63<br>3×7=21, 5×9=45	4×7=28, 8×9=72<br>4×6=24, 6×7=42
9	7×8=56, 8×9=72<br>4×6=24, 5×9=45	3×8=24, 6×8=48<br>6×7=42, 4×8=32	4×7=28, 6×9=54<br>3×6=18, 5×7=35	3×9=27, 7×9=63<br>3×7=21, 4×9=36
11	4×7=28, 6×8=48<br>4×6=24, 6×7=42	3×8=24, 5×9=45<br>3×7=21, 7×8=56	4×9=36, 8×9=72<br>3×9=27, 7×9=63	5×7=35, 6×9=54<br>3×6=18, 4×8=32
12	7×8=56, 8×9=72<br>3×6=18, 5×9=45	3×8=24, 6×8=48<br>6×7=42, 4×8=32	4×7=28, 6×9=54<br>4×6=24, 5×7=35	3×9=27, 7×9=63<br>3×7=21, 4×9=36
13	6×7=42, 6×8=48<br>4×7=28, 3×8=24	7×8=56, 5×9=45<br>4×6=24, 4×9=36	5×7=35, 7×9=63<br>3×7=21, 3×9=27	4×8=32, 8×9=72<br>3×6=18, 6×9=54
16	5×7=35, 7×9=63<br>3×7=21, 3×9=27	7×8=56, 5×9=45<br>3×8=24, 4×9=36	4×7=28, 6×8=48<br>4×6=24, 6×7=42	4×8=32, 8×9=72<br>3×6=18, 6×9=54
19	7×8=56, 6×9=54<br>3×6=18, 3×9=27	3×8=24, 4×9=36<br>3×7=21, 4×8=32	5×9=45, 8×9=72<br>5×7=35, 7×9=63	4×7=28, 6×8=48<br>4×6=24, 6×7=42
23	4×7=28, 6×8=48<br>4×6=24, 6×7=42	5×7=35, 6×9=54<br>3×6=18, 3×8=24	4×9=36, 8×9=72<br>3×9=27, 7×9=63	4×8=32, 5×9=45<br>3×7=21, 7×8=56
25	3×9=27, 8×9=72<br>3×7=21, 7×9=63	7×8=56, 5×9=45<br>3×8=24, 4×9=36	4×7=28, 4×8=32<br>4×6=24, 6×7=42	5×7=35, 6×9=54<br>3×6=18, 6×8=48
27	5×7=35, 8×9=72<br>3×6=18, 6×8=48	4×7=28, 3×9=27<br>3×7=21, 3×8=24	7×8=56, 7×9=63<br>4×8=32, 5×9=45	6×7=42, 6×9=54<br>4×6=24, 4×9=36
31	4×8=32, 4×9=36<br>3×8=24, 3×9=27	3×7=21, 6×9=54<br>3×6=18, 5×7=35	4×7=28, 8×9=72<br>4×6=24, 6×7=42	7×8=56, 7×9=63<br>6×8=48, 5×9=45
33	7×8=56, 5×9=45<br>3×7=21, 4×9=36	4×7=28, 6×8=48<br>4×6=24, 6×7=42	5×7=35, 8×9=72<br>3×6=18, 6×9=54	4×8=32, 7×9=63<br>3×8=24, 3×9=27
34	5×9=45, 7×9=63<br>4×9=36, 6×9=54	3×7=21, 8×9=72<br>4×6=24, 7×8=56	3×8=24, 6×8=48<br>6×7=42, 4×8=32	4×7=28, 3×9=27<br>3×6=18, 5×7=35

Table S2. Similarity and problem size of the trained sets for the excluded participants (same kind of information as Table 1 in the main text).

	Similarity in…		Average operands size in…	
Participant	Low-similarity sets	High-similarity sets	Low-similarity sets	High-similarity sets
3	2, 2	12, 12	6.44	6.31
5	2, 2	11, 11	6.38	6.38
8	2, 2	11, 11	6.0	6.75
14	1, 1	10, 11	6.13	6.63
15	1, 1	10, 10	6.44	6.31
17	1, 1	9, 9	6.13	6.63
18	1, 1	10, 10	6.75	6.0
20	2, 2	11, 11	6.38	6.38
21	2, 2	11, 11	6.0	6.75
22	1, 1	10, 10	6.44	6.31
24	1, 1	9, 9	6.13	6.63
26	0, 1	10, 11	6.56	6.19
28	2, 2	11, 11	6.56	6.19
29	1, 1	11, 13	6.50	6.25
30	2, 2	11, 11	6.63	6.13
32	1, 1	9, 9	6.13	6.63
35	2, 2	11, 11	6.56	6.19
Average	1.44	10.53	6.36	6.39

2. Participant exclusion

Table S3. The list of participants who did not finish the experiment (and were not included in the data analysis), and the reason for excluding each participant.

ID	Quit after…	Exclusion reason
26	Pre-experiment test	Pre-existing multiplication knowledge
30	Pre-experiment test	Pre-existing multiplication knowledge
31	Pre-experiment test	Pre-existing multiplication knowledge
24	Pre-experiment test	Inattentive
35	Post-experiment test	The child decided to quit, after being uncooperative during the experiment.
28	Week 1	The child decided to quit
21	Week 1	Not following the experiment rules.
10	Week 1	Not cooperative
3	Week 1	Inattentive, repetition errors
22	Week 2	Not cooperative
15	Week 2	Inattentive
20	Week 2	Inattentive, repetition errors
5	Week 3	Not cooperative, inattentive
17	Week 3	Inattentive, repetition errors
32	Week 3	Inattentive, repetition errors
29	Week 3	Inattentive
18	Week 4	The child decided to quit
14	Week 4	Not cooperative, Inattentive, repetition errors

Exclusion reasons:

- *Pre-existing multiplication knowledge*: the child answered correctly to at least one of the 16 facts to be learned in the experiment. “Correct” was defined as providing the correct answer to a particular fact at least twice (out of the 3 attempts).
- The child decided to quit.
- *Not following the experiment rules*: the parents of one participant provided help by teaching multiplication strategies, in contrast to the experiment instructions.
- *Not cooperative* means that according to the experimenter’s best judgment, the child showed low motivation and was uninterested in the experiment to the degree that may impair performance.
- *Inattentiveness* means that according to the experimenter’s best judgment, the participant was extremely inattentive, to the degree of being unable to focus on the task effectively.

- *Repetition errors* is an objective criterion for inattentiveness. It means that the participant made more than 15 repetition errors in at least one training day (Fig. S1). A "repetition error" refers to the first stage of each training round, in which the participant repeated the facts said by experimenter. Note that this criterion does not confound with the child's performance, and critically it does not confound with the effect of similarity, because the criterion is based on the children's ability to repeat the facts said by experimenter in the first part of each training round, whereas our measures of learning progress were based on the children's retrieval of facts in the second part of each training round.

  The 15-errors threshold was not exceeded by any of the participants who remained in the study, except one participant (#25) in the first training day.

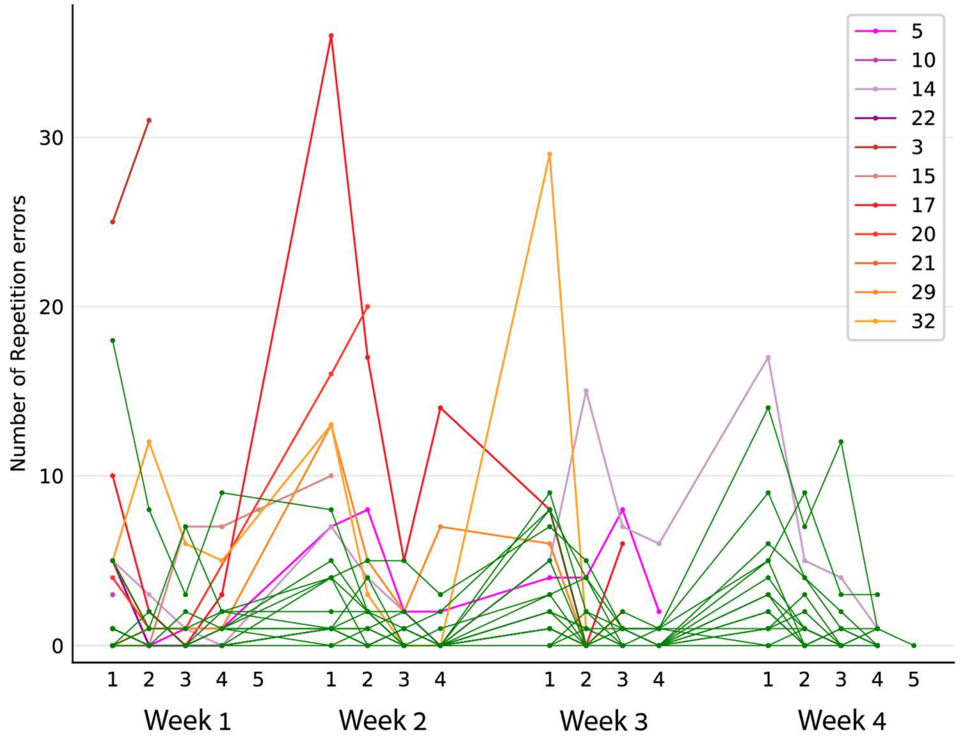

Fig. S1. The total number of repetition errors, i.e., repeating incorrectly a fact said by the experimenter, in each training day. Each line represents one participant, the legend shows participant numbers. Color codes: green = participants in the study; pink = excluded for being uncooperative; red/orange = excluded for inattentiveness.

3. Participant exclusion in Experiment 2

This data refers to the control experiment in which children performed only the forced-choice test, with no preceding learning. We excluded children with stereotypical responses – i.e., children who consistently chose the second alternative presented to them (no child consistently chose the first alternative). Table S4 shows the rate of trials in which each child chose the second alternative.

Table S4. The rate of trials in which a participant chose the second alternative in the forced-choice control experiment

Participant	Alternative #2	
14	25%	
30	34%	
26	41%	
1	48%	
13	50%	
18	53%	
9	55%	
2	56%	
16	59%	
27	63%	
4	64%	
6	66%	
31	66%	
10	69%	
17	69%	
29	84%	Excluded
21	88%	Excluded
8	91%	Excluded

4. Deviations from the experiment protocol

In a small number of cases, the experiment slightly deviated from the standard training protocol. We hereby detail these cases and how they were addressed:

- **Learned exercise.** Participant #19 started learning the exercise 3×6 in class during the study period. This specific exercise was therefore excluded from all analyses for that participant.
- **Inattentive participant.** One participant (#16) was extremely inattentive in two sessions in the 4th training week. To compensate for this, we added a 5th training day for that participant during that particular week.
- **Human error.** The experiment protocol dictated that when asking the children to retrieve the multiplication facts during the training rounds, the experimenter first asked the child to say any fact they remembered; then, if the child completely failed to mention some facts, the experimenter presented that fact and asked what the answer was. In very few cases, due to human error, the experimenters did the first part of the protocol, but did not follow to the second part – i.e., they did not present the facts that the child completely forgot. This was the case for 5 participants (#1, #4, #6, #12, #16), in the first 3 days of the first training week (for participant #1, only in the first day of the first week). To compensate for this, we added a 5th training day to these children during the first week.

  We verified that this minor breach of protocol did not affect the results. First, of the 5 children, the first week included high-similarity facts for 3 children (#1, #6, #16) and low-similarity facts for the other two. Thus, the experimenter mistake was equally distributed across low-similarity and high-similarity sets. Second, as Figure 2a clearly shows, the mistake did not affect the per-child end-of-week results.

In the analyses that examined the performance separately for each day, we considered only the first four days of each week and ignored the fifth training day, if it existed.

5. Pre-experiment addition and subtraction test

In this test, ran in the first day of the study (in week 1), each child solved 10 addition and 7 subtraction exercises. The average accuracy was 71.2% in the addition exercises and 53.8% in the subtraction exercises. This difference is significant (t(16) = 3.35, one-tailed p = .002), however, note that the additions and subtractions were not balanced. Table S5 shows the average accuracy for each exercise. Table S6 shows the average accuracy for each participant; unsurprisingly, there was much variance between participants. The full per-exercise, per-participant information is available in the "tests.csv" file in the supplementary data repository.

Table S5. Accuracy in each addition and subtraction exercise in the pre-experiment tests in week 1

Exercise	Accuracy	Exercise	Accuracy
2+4	82%	5-3	71%
2+5	76%	8-5	53%
2+9	76%	9-3	65%
3+2	71%	9-6	41%
3+6	76%	10-4	59%
4+3	71%	12-4	65%
5+3	76%	14-6	24%
5+4	76%		
6+6	76%		
8+7	29%		

Table S6. Addition/subtraction accuracy per participant in the pre-experiment tests in week 1

Participant	Accuracy in addition exercises	Accuracy in subtraction exercises
1	100%	57%
2	100%	86%
4	70%	43%
6	20%	14%
7	90%	43%
9	80%	57%
11	60%	0%
12	90%	100%
13	100%	100%
16	40%	43%
19	80%	86%
23	100%	86%
25	20%	0%
27	70%	86%
31	20%	0%
33	70%	29%
34	100%	86%

6. Accuracy and similarity effect in the 2nd testing round in the weekly tests

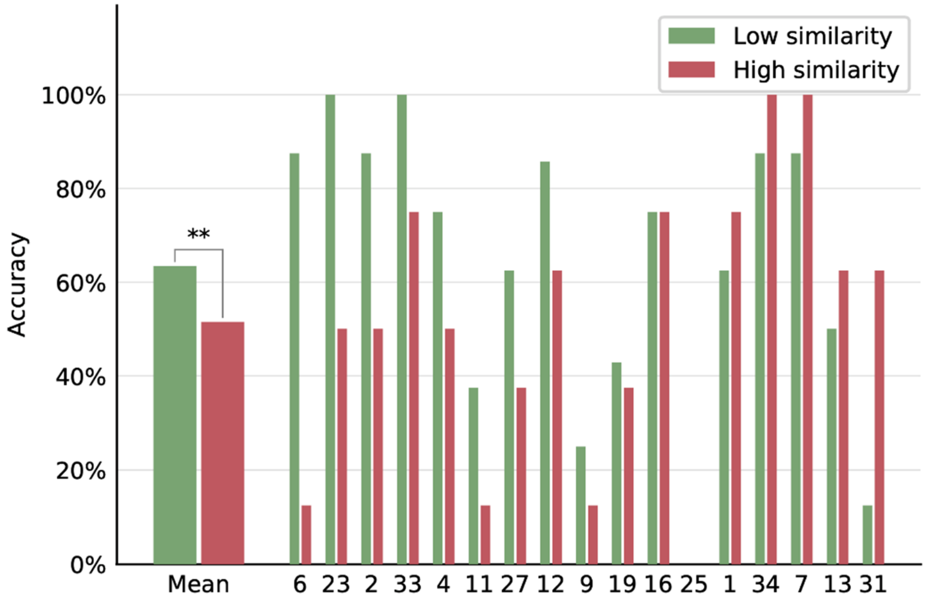

Fig. S2. In the second round of the end-of-week tests, the effect of similarity could still be observed. The effect size was similar to the first round, but with lower significance levels.

7. Performance in each fact

All participants were trained on the same 16 facts. In the pre-experiment and post-experiment tests, they were tested on these facts, but also on 27 facts for which they received no training. These included 7 rule-based facts (products of 0 or 1: 0×2, 0×3, 0×6, 1×4, 1×5, 1×8, 1×9), and 20 facts that presumably require explicit learning (2×2, 2×3, 2×4, 2×5, 2×6, 2×7, 2×8, 2×9, 3×3, 3×4, 3×5, 4×4, 4×5, 5×5, 5×6, 5×8, 6×6, 7×7, 8×8, 9×9). Unsurprisingly, the children performed better in the *untrained* facts, which were much easier than the trained facts (Fig. S3). Tables S7, S8 show the performance in each of the facts on which the children were tested.

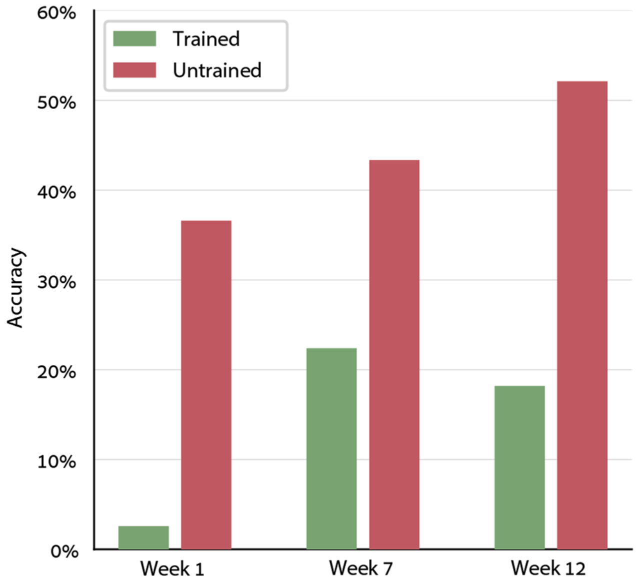

Fig. S3. Accuracy in the trained versus untrained facts in the pre-experiment (week 1) and post-experiment (weeks 7, 12) recall tests.

Table S7. Accuracy in each trained fact

Fact	Pre-experiment	Weekly test	Post-experiment		Forced choice		
			Week 7	Week 12	Week 7	Week 12 round 1	Week 12 round 2
3×6=18	6%	81%	31%	40%	88%	88%	76%
3×7=21	4%	82%	24%	20%	69%	53%	88%
3×8=24	2%	65%	8%	12%	47%	47%	41%
3×9=27	2%	76%	24%	16%	47%	47%	44%
4×6=24	6%	71%	27%	24%	59%	65%	59%
4×7=28	2%	65%	29%	12%	59%	65%	53%
4×8=32	2%	29%	16%	6%	47%	59%	71%
4×9=36	2%	59%	20%	10%	24%	53%	41%
5×7=35	2%	53%	24%	18%	29%	53%	59%
5×9=45	2%	59%	18%	18%	50%	59%	65%
6×7=42	2%	53%	24%	14%	65%	65%	35%
6×8=48	2%	41%	18%	18%	65%	53%	59%
6×9=54	0%	59%	18%	18%	59%	59%	65%
7×8=56	2%	63%	16%	18%	38%	44%	81%
7×9=63	2%	65%	27%	27%	81%	65%	53%
8×9=72	2%	82%	37%	25%	75%	76%	65%

Table S8. Accuracy in each untrained fact

		Post-experiment				Post-experiment	
Fact	Pre-experiment	Week 7	Week 12	Fact	Pre-experiment	Week 7	Week 12
2×2=4	75%	84%	84%	0×2=0	67%	78%	88%
2×3=6	51%	57%	67%	0×3=0	55%	73%	88%
2×4=8	45%	61%	78%	0×6=0	63%	76%	80%
2×5=10	53%	51%	69%	1×4=4	76%	75%	90%
2×6=12	39%	47%	53%	1×5=5	71%	78%	86%
2×7=14	37%	43%	54%	1×8=8	73%	76%	82%
2×8=16	33%	47%	52%	1×9=9	75%	76%	90%
2×9=18	20%	41%	51%				
3×3=9	43%	50%	66%				
3×4=12	14%	25%	39%				
3×5=15	27%	37%	52%				
4×4=16	22%	25%	32%				
4×5=20	14%	32%	39%				
5×5=25	24%	20%	37%				
5×6=30	2%	6%	10%				
5×8=40	2%	0%	4%				
6×6=36	4%	4%	6%				
7×7=49	2%	0%	2%				
8×8=64	2%	0%	2%				
9×9=81	2%	6%	4%				

8. Detailed results of linear mixed models

The following tables include the full details of each logistic linear mixed model described in the main text. In all these models, the dependent variable was the accuracy in each fact.

8.1 Analyses from Section 3.1: Learning dissimilar facts is easier

Table S9a. The effect of Numeric Similarity on accuracy in the weekly tests, round 1

Factor	Odds ratio	95% confidence interval
Intercept	39.26	0.03 – 48,227
Numeric similarity	0.43	0.25 – 0.74
Product of operands	1.02	0.89 – 1.17
Sum of operands	0.78	0.30 – 2.06
Random effects		
σ^2 within participant & exercise	3.29	
σ^2 between participants	1.47	
σ^2 between exercises	0.36	

Table S9b. The effect of Similarity Level on accuracy in the weekly tests, round 1

Factor	Odds ratio	95% confidence interval
Intercept	71.22	0.08 – 61,261
Similarity level = High	0.41	0.21 – 0.80
Product of operands	1.04	0.92 – 1.19
Sum of operands	0.69	0.28 – 1.72
Random effects		
σ^2 within participant & exercise	3.29	
σ^2 between participants	1.41	
σ^2 between exercises	0.30	

Table S10a. The effect of Numeric Similarity on accuracy in the weekly tests, round 2

Factor	Odds ratio	95% confidence interval
Intercept	2,310	1.36 – 3,929,193
Numeric similarity	0.49	0.29 – 0.84
Product of operands	1.12	0.97 – 1.29
Sum of operands	0.42	0.15 – 1.14
Random effects		
σ^2 within participant & exercise	3.29	
σ^2 between participants	2.13	
σ^2 between exercises	0.39	

Table S10b. The effect of Similarity Level on accuracy in the weekly tests, round 2

Factor	Odds ratio	95% confidence interval
Intercept	4,153	1.75 – 9,853,421
Similarity level = High	0.58	0.30 – 1.13
Product of operands	1.14	0.99 – 1.33
Sum of operands	0.36	0.13 – 1.04
Random effects		
σ^2 within participant & exercise	3.29	
σ^2 between participants	2.07	
σ^2 between exercises	0.47	

8.2 Analyses from Section 3.2: The similarity effect arises from the grouping of facts

Table S11. The effect of Numeric Similarity on top of Similarity Level in the weekly tests, round 1

Factor	Odds ratio	95% confidence interval
Intercept	38.86	0.03 – 48,477
Numeric similarity	0.42	0.16 – 1.12
Similarity level = High	1.03	0.30 – 3.48
Product of operands	1.02	0.89 – 1.17
Sum of operands	0.78	0.30 – 2.06
Random effects		
σ^2 within participant & exercise	3.29	
σ^2 between participants	1.47	
σ^2 between exercises	0.36	

8.3 Analyses from Section 3.3: Similarity affects long-term memory directly

Table S12. Similarity effect in the pre-session test of each training session

	Day 2		Day 3		Day 4	
Factor	Odds ratio	95% CI	Odds ratio	95% CI	Odds ratio	95% CI
Intercept	0.14	0.06 – 0.36	19.36	0.03 – 12,469	299.45	0.82 – 109,910
Numeric similarity	1.01	0.68 – 1.50	0.97	0.60 – 1.59	0.53	0.32 – 0.87
Product of operands	13.70	1.25 – 149.73	1.05	0.92 – 1.19	1.06	0.95 – 1.19
Sum of operands	0.08	0.01 – 0.84	0.65	0.27 – 1.57	0.57	0.26 – 1.26
Random effects						
σ^2 within	3.29		3.29		3.29	
σ^2 between participants	1.97		1.20		1.92	
σ^2 between exercises	0.30		0.29		0.14	

Table S13. Similarity effect in the pre-session test of each training session: interaction between similarity and 4th day versus the preceding days

Factor	Odds ratio	95% confidence interval
Intercept	0.28	0.14 – 0.56
Numeric similarity	0.96	0.74 – 1.24
Day = 4	4.92	3.37 – 7.18
Similarity × Day=4 interaction	0.68	0.47 – 0.98
Product of operands	3.38	0.60 – 19.15
Sum of operands	0.25	0.04 – 1.39
Random effects		
σ^2 within participant & exercise	3.29	
σ^2 between participants	1.54	
σ^2 between exercises	0.35	

Table S14. Similarity effect on the response attempts during training

	Day 1		Day 2		Day 3		Day 4	
Factor	Odds ratio	95% CI	Odds ratio	95% CI	Odds ratio	95% CI	Odds ratio	95% CI
Intercept	2.05	0.07 – 57	18.52	0.29 – 1,186	1.66	0.03 – 88.9	206.3	1.02 – 41,780
Numeric similarity	0.94	0.73 – 1.22	1.05	0.81 – 1.36	1.02	0.78 – 1.34	0.70	0.52 – 0.93
Round (1-4)	1.25	1.10 – 1.42	1.18	1.04 – 1.34	1.25	1.09 – 1.42	1.11	0.96 – 1.28
Position within round	1.80	1.58 – 2.05	1.84	1.61 – 2.11	1.59	1.39 – 1.82	1.54	1.33 – 1.78
Product of operands	1.06	0.99 – 1.13	1.09	1.01 – 1.18	1.05	0.98 – 1.13	1.10	1.00 – 1.21
Sum of operands	0.64	0.41 – 1.00	0.50	0.29 – 0.87	0.67	0.40 – 1.10	0.46	0.24 – 0.89
Random effects σ^2								
Within	3.29		3.29		3.29		3.29	
Between participants	0.40		1.34		1.87		2.15	
Between exercises	0.07		0.15		0.11		0.22	

Table S15. The effect of numeric similarity on the 2nd round of forced-choice test in week 12

Factor	Odds ratio	95% confidence interval
Intercept	1.52	1.16 – 1.98
Product of operands	2.53	0.68 – 9.46
Sum of operands	0.36	0.10 – 1.36
Numeric similarity	0.67	0.52 – 0.87
Random effects		
σ^2 within participant	3.29	
σ^2 between participants	0.03	
